# lncRNA ZFAS1 Promotes HMGCR mRNA Stabilization via Binding U2AF2 to Modulate Pancreatic Carcinoma Lipometabolism

**DOI:** 10.1155/2022/4163198

**Published:** 2022-07-08

**Authors:** Luoluo Wang, Yi Ruan, Xiang Wu, Xinhua Zhou

**Affiliations:** Department of Minimal Invasive Surgery, Ningbo Medical Center Lihuili Hospital, Ningbo, 315000 Zhejiang, China

## Abstract

Being one of the most lethal malignant tumors worldwide, pancreatic carcinoma (PC) shows strong invasiveness and high mortality. In tumorigenesis and progression, the role played by long-chain noncoding RNAs (lncRNAs) cannot be ignored. This article mainly probes into the function of lncRNA ZFAS1 in PC. ZFAS1 expression in PC and normal counterparts retrieved from the Genotype-Tissue Expression (GTEx) project and The Cancer Genome Atlas (TCGA) database was analysed by GEPIA2. Its expression profile in clinical specimens and human PC cell strains was quantified using qRT-PCR. Measurements of BxPC-3 cell multiplication and invasiveness employed CCK-8, plate clone formation test, and Transwell chamber assay. ZFAS1's impact on lipid content in BxPC-3 cells was detected. RNA pulldown and RIP assays analyzed the interaction of ZFAS1 with U2AF2 and HMGCR in BxPC-3 cells. Finally, the impacts of U2AF2 and HMGCR on the biological behavior of BxPC-3 were observed. ZFAS1 was kept at a high level in PC tissues versus the normal counterparts. ZFAS1 gene knockout remarkably suppressed PC cell multiplication and invasiveness and decreased the contents of free fatty acids, total cholesterol, triglycerides, and phospholipids. Mechanistically, ZFAS1 stabilized HMGCR mRNA through U2AF2, thus increasing HMGCR expression and promoting PC lipid accumulation. Meanwhile, reduced PC cell viability and invasiveness were observed after downregulating U2AF2 and HMGCR. As an oncogene of PC, ZFAS1 can modulate lipometabolism and stabilize HMGCR mRNA expression by binding with U2AF2 in PC, which is a candidate target for PC diagnosis and treatment.

## 1. Introduction

With a five-year survival under 10%, pancreatic carcinoma (PC) is an extremely fatal and malignant digestive tract tumor [1]. Surgical resection is the most effective means for PC treatment. Nonetheless, due to inconspicuous early symptoms, the disease has frequently advanced locally to arteriovenous invasiveness and/or early distant metastasis, leaving only a small percentage (5-10%) of patients with tumors that can be surgically removed with a chance of cure [2–4]. And despite the relatively effective treatment, fewer than 4% survive for ten years or more [5]. Given the current situation of PC treatment, it is urgent to clarify the potential molecular mechanisms of PC and work on finding feasible biomarkers and therapeutic targets to provide tailored medical services and enhance patient outcomes.

Recently, mass evidence has demonstrated the strong connection between lipometabolism disorders and tumor cells' malignant biological behavior [6, 7]. Lipids are essential in maintaining normal cell function and homeostasis. In addition to their critical roles as important components of cell membranes, they provide precursors for vital molecules involved in the pathways responsible for growth and differentiation [8, 9]. One of the most distinctive metabolic abnormalities among cancer-associated disorders is dysregulation of lipometabolism [10]. Cancer cells use lipometabolism to get energy, biofilm components, and signal molecules required to proliferate, survive, invade, metastasize, and respond to tumor microenvironment and cancer treatment [11]. Lipids can sufficiently stimulate PC cell multiplication [12]. Lipogenic enzymes are often overexpressed in various cancers, including PC [13–15]. Therefore, finding a reliable molecule involved in lipometabolism regulation and OC progression is also pressing. Long-chain noncoding RNAs (lncRNAs) longer than 200 nucleotides are essentially a kind of ncRNAs with diverse and nonspecific biological functions.

In recent years, lncRNAs have been revealed as key regulators in tumorigenesis [16]. Meanwhile, mass studies have shown their aberrant expression in PC, participating in biological processes such as multiplication, metastasis, and chemotherapy resistance [17–19]. lncRNAs generally play different roles in cytoplasm and nucleus [20]. In mammals, it is generally believed that the glucose-fatty acid- (FA-) protein metabolism balance is essential, and the break of the balance can induce various diseases and even tumors [21]. A large amount of evidence shows that in various diseases, especially during cell carcinogenesis, metabolic patterns involving mitochondrial oxidative phosphorylation, glycolysis, and FA oxidation, a phenomenon researchers called tumor cell metabolic reprogramming, alter greatly [22, 23]. In tumor cells, many key enzymes associated with lipolysis and lipid synthesis are over-expressed [24]. lncRNAs have been reported to interfere with lymph node metastasis of cervical cancer, which promote FA metabolism reprogramming and cervical cancer cell metastasis via modulating FABP5—a carrier for FA uptake and transport [25].

Located on chromosome 20q13. 13, ZNFX1 Antisense RNA 1 (ZFAS1) is a transcript of the encoding gene identified as a regulator of breast alveolar and epithelial cell differentiation [26]. ZFAS1 was initially reduced in breast cancer tissue, suggesting that this transcript has tumor-suppressive effects [27]. Subsequent studies, however, report that ZFAS1 exerts an opposite function during the progression of most cancer types. Besides its role in tumor pathogenesis, ZFAS1 is involved in the molecular cascade leading to a series of diseases, such as osteoarthritis [28], epilepsy [29], and atherosclerosis [30]. But the molecular mechanism ZFAS1 participates in metabolic reprogramming remains uncharacterized. Precursor messenger RNA (pre-mRNA) splicing is a key step in processing gene transcripts that encode most eukaryotic proteins and is a part of a series of ordered reactions catalyzed by large RNA-protein complexes called spliceosomes [31–33]. Alternative splicing may generate different mRNAs and proteins from individual transcript, which is critical in development, differentiation, and multiple human diseases, including cancer [34, 35]. The U2 Auxiliary Factor complex, one of the components of spliceosomes, comprises a small (U2AF1) and a large (U2AF2) subunit. As essential proteins, U2AF1 and U2AF2 work together to bind U2 snRNP to the 3 splice site of introns in most eukaryotes [36]. Recently, U2AF1 somatic mutations have been repeatedly demonstrated in a number of human tumors [37, 38]. HMGCR, abbreviated from 3-hydroxy-3-methyl-glutarylcoenzyme-A reductase, is a rate-limiting enzyme of cholesterol (CHOL) biosynthesis and has been found to have carcinogenic effects in gastric cancer, glioblastoma, and prostate cells. Based on the above information, the novelty and motivation of this paper is to explore ZFAS1 expression and its mechanism in cases with PC and try to clarify whether ZFAS1 can influence PC progression through lipometabolism for the first time.

## 2. Materials and Methods

### 2.1. Clinical Specimen Collection

Between February 2020 and December 2021, 43 paired PC and adjacent equivalents specimens were collected from PC patients who received no preoperative chemoradiotherapy but surgical treatment at the Department of General Surgery of Ningbo Medical Center Lihuili Hospital. PC tissues and normal counterparts resected intraoperatively were isolated for immediate liquid nitrogen storage until analysis. All postoperative specimens were pathologically confirmed, and all patients signed informed consent, agreeing to donate pancreas and PC tissues for relevant experimental detection. Our hospital has ethically ratified this research. The Gene Expression Profiling Interactive Analysis (GEPIA2) public database (https://gepia2.cancer-pku.cn/ index) was responsible for analyzing related molecule expression in PC specimens in The Cancer Genome Atlas (TCGA) database (https://portal.gdc.cancer.gov/).

### 2.2. Cell Cultivation, Treatment, and Cytoplasmic and Nuclear RNA Isolation

Supplied by Shanghai Institutes for Biological Sciences, Chinese Academy of Sciences (CAS) cell bank, BxPC-3, PANC-1, SW1990, PaCa-2 (human PC cell strains), and HPNE (human pancreatic ductal epithelial cells) were conventionally cultivated by immersing in DMEM (Gibco, USA)+10% fetal bovine serum (FBS, Thermo Fisher Scientific, USA) in a 37°C incubator (5% CO_2_). GenePharma (Shanghai, China) was responsible for designing and synthesizing the siRNA targeting ZFAS1, U2AF2, and HMGCR and the negative control si-NC. On the day before transfection, logarithmic growth-phase BxPC-3 cells were seeded into the wells of a 6-well plate and for siRNA and si-NC transfection following the manufacturer's instructions of Lipofectamine 3000 (Invitrogen, USA) when the cell density reached 40%.

### 2.3. Cytoplasmic and Nuclear RNA Isolation

ZFAS1 distribution in BxPC-3 cell's cytoplasm and nucleus was determined by referring to the PARIS kit recommendations (Life Technology, USA) by referring to the supplier's manuals. In general, cells after being washed with PBS were lysed and centrifuged (300 g, 5 min); separate the supernatant (cytoplasm) and remaining pellet (nuclear) after being washed by PBS 5 times.

### 2.4. qRT-PCR

After being isolated using TRIzol (Invitrogen, USA), cell and tissue total RNA were reverse-transcribed using a PrimeScript RT Reagent Kit (Takara, Japan). Following that, using the ABI Step One Real-time PCR System (Thermo Fisher Scientific, USA), a PCR reaction was performed on the cDNA according to the SYBR Green Kit recommendations (Takara, Japan). Shanghai Sangon synthesized the specific primers (Table 1) used in qRT-PCR. lncRNA ZFAS1 expression and U2AF2 and HMGCR expression normalized with *β*-actin and GAPDH, respectively, were computed using 2^-*ΔΔ*Ct^. The average value was obtained after running the experiment three times.

### 2.5. Western Blotting (WB)

The total protein tested RIPA protein lysate and PMSF protease inhibitor extracted total protein for concentration determination. Then, 20 *μ*g total protein was sampled and separated on SDS-PAGE with a concentration of 10%, followed by transfer onto a PVDF membrane, 1 h of 5% defatted milk blocking, as well as overnight immersion (4°C) in corresponding primary antibodies U2AF2 (1 : 1000, Cell Signaling Technology, USA), HMGCR (1 *μ*g/mL, Abcam, UK), and *β*-actin (1 : 1000, Abcam, UK). After washing the membrane, horseradish peroxidase-labeled goat anti-rabbit IgG (Abcam, UK) diluted at 1 : 2000 was added for reaction (37°C, 1 h). Following another membrane rinsing, development was conducted using an ECL kit (Millipore, NY), and an analysis of protein bands' gray values was performed via ImageJ software (version 1.8.0; National Institutes of Health).

### 2.6. Cell Multiplication Test

Cell multiplication determination was performed by cell counting kit-8 (CCK-8) (Dojindo, Japan). Following a 24-hour transfection, cells (1 × 10^3^/well) were cultured in the wells of a 96-well plate for 24, 48, 72, and 96 hours (37°C, 5% CO_2_). After this, CCK-8 solution (10 *μ*L) was dripped into each well for additional 2 hours of culture. Finally, absorbance determination was conducted using a microplate reader (Bio-Rad, USA), and the measuring wavelength indicating cell multiplication was 450 nm. The experiment was repeatedly determined three times at each time point and averaged the value.

### 2.7. Cell Clone Formation Experiment

Hep-2 cells (1 × 10^3^/well) transfected for 24 hours were inoculated in the wells of a 6-well plate, after which they were immersed in a complete medium for 2 weeks of cultivation. Subsequently, the colonies with ≥50 cells were counted by microscope (Nikon, Japan) after cell fixation by 4% paraformaldehyde as well as staining via 0.1% crystal violet (CV).

### 2.8. Transwell

Cell invasiveness capacity was tested using the Transwell cell invasion assay. First, Matrigel matrix glue was synthesized and placed into the upper chamber for incubation (37°C, 2 hours). The transfected cells were routinely digested and suspended in a serum-free medium post a 24-hour culture. They were inoculated in the upper chamber with 2 × 10^4^ cells/well following cell density adjustment. The culture solution comprising 10% FBS was placed in the basolateral chamber, while the cells in the apical chamber were subjected to the removal with Q-tips, two PBS rinses, 4% paraformaldehyde immobilization, and 0.1% CV dying after 24 h of conventional culture for the final cell counting via microscopical photographing.

### 2.9. Lipid Assays

EnzyChrom free fatty acid (FFA), EnzyChrom CHOL, EnzyChrom Triglyceride, and EnzyChrom Phospholipid assay kits, all provided by BioAssay Systems, USA, were used following the corresponding supplier's instructions to measure the contents of FFAs, total cholesterol (TC), triglycerides (TGs), and phospholipids (PLs) in cells.

### 2.10. RNA Pulldown Assay

The biotin-labeled RNA pulldown assay was conducted via the Pierce™ magnetic RNA-protein pulldown kit supplied by Thermo Fisher Scientific, USA. RNeasy Plus Mini Kit and DNase I (Qiagen, Germany) were used to obtain a biotin-labeled RNA transcript, which was then mixed with resuspended streptavidin-labeled magnetic beads and incubated overnight (4°C) after in vitro transcription of ZFAS1 using T7RNA polymerase (Ambio life, USA). Then, they were incubated indoor in lysate for 1 h, together with the RNase inhibitor. WB was carried out after bead washing with a wash buffer to determine the eluted proteins.

### 2.11. RNA-Binding Protein Immunoprecipitation (RIP) Assay

Immunoprecipitation analysis of RNA binding proteins was made by referring to the EZ-Magna RIP Kit (Millipore USA) instructions. Cells were treated with cold PBS rinsing and cleavage with RIP lysis buffer. U2AF2 antibody (1 : 50, Cell Signaling, USA) and nonspecific control antibody IgG were used for immunoprecipitation. RIP lysate was incubated with a magnetic bead binding antibody overnight (4°C). Following that, proteinase K was used for immunoprecipitated protein degradation. The bound RNA was separated from the supernatant, and the RNA concentration was measured using NanoDrop (Thermo Fisher Scientific, USA). Furthermore, the purified RNA was analyzed by RT-qPCR to detect ZFAS1 and HMGCR mRNA levels.

### 2.12. Stability Analysis of RNA

BxPC-3 cells were treated with 6 and 12 h of incubation with 2.5 mg/mL actinomycin D (ActD) after 48 h of transfection, while those without ActD intervention served as the negative control. HMGCR mRNA stabilization was detected by qRT-PCR after isolating the total RNA.

### 2.13. Statistical Processing

SPSS 22.0 was adopted for data analysis and GraphPad prism 8.0 for rendering and presentation. Quantitative variables were given as mean ± standard deviation (Mean ± SD), and double-tailed *P* < 0.05 was deemed statistically significant. Each assay was repeatedly determined 3 times. The difference in ZFAS1 expression between PC patients and normal controls was identified using the nonparametric Mann–Whitney *U* test. The comparison methods for quantitative variables were independent sample *t*-test (intergroup) and one-way ANOVA plus Bonferroni posttest (multigroup).

## 3. Results

### 3.1. ZFAS1 Keeps at a Higher Level in PC and PC Cells

**Figure 1:**
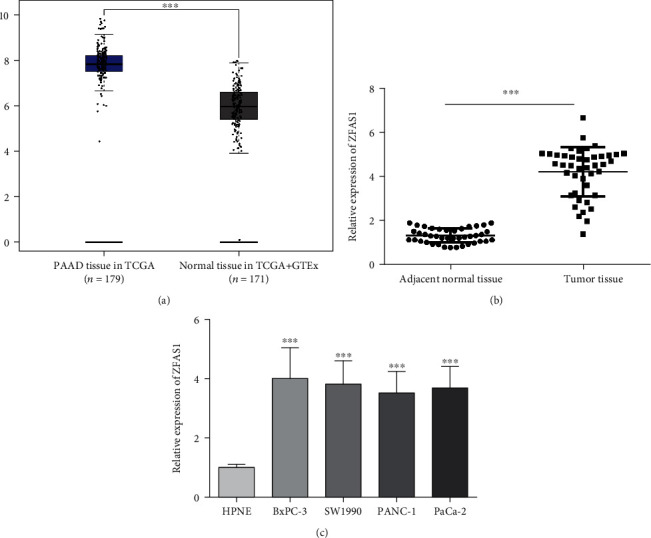
ZFAS1 in PC. (a) ZFAS1 expression in TCGA+GTEx database samples; (b) ZFAS1 expression in clinical samples; (c) ZFAS1 in different cell strains; ^∗∗∗^*P* < 0.001*vs.* HPNE group.

We first analyzed PAAD transcriptome data from TCGA database using GEPIA2, combined with its expression data in normal tissue from the GTEx database and found that ZFAS1 was kept at a higher level in PC (Figure 1(a)). Subsequently, we used qRT-PCR to analyze ZFAS1 in 43 PC tissue specimens and normal counterparts and found notably higher ZFAS1 in cancerous tissue specimens (*P* < 0.05, Figure 1(b)). Furthermore, upregulated ZFAS1 was determined in human PC cell strains (BxPC-3, PANC-1, SW1990, and PaCa-2) versus HPNE (*P* < 0.05, Figure 1(c)).

### 3.2. Impact of ZFAS1 Knockout on PC Cell Biological Function

**Figure 2:**
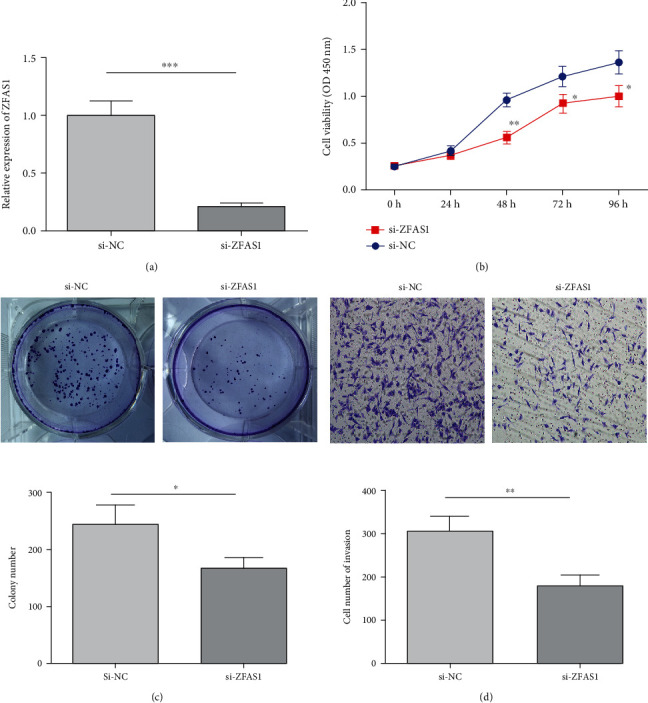
Impact of ZFAS1 knockout on biological function of PC cells. (a) ZFAS1 expression; (b) cell viability; (c) plate clonal cell formation; (d) cell invasiveness; ^∗^*P* < 0.05;  ^∗∗^*P* < 0.01;  ^∗∗∗^*P* < 0.001.

To study ZFAS1's biological function in PC, ZFAS1 siRNA transfection into BxPC-3 cells was conducted, only to find notably decreased ZFAS1 in BxPC-3cells following transfection (*P* < 0.05, Figure 2(a)). We also observed notably suppressed BxPC-3 viability (*P* < 0.05, Figure 2(b)) and statistically reduced clone formation number (*P* < 0.05, Figure 2(c)) after si-ZFAS1 transfection, as indicated by CCK-8 and plate-clone formation experiments. The Transwell test showed weakened invasiveness of BxPC-3 cells post-si-ZFAS1 transfection (*P* < 0.05, Figure 2(d)). Thus, ZFAS1 promotes PC cell multiplication and invasiveness.

### 3.3. ZFAS1 Participates in PC Cell Lipometabolism via Modulating HMGCR

**Figure 3:**
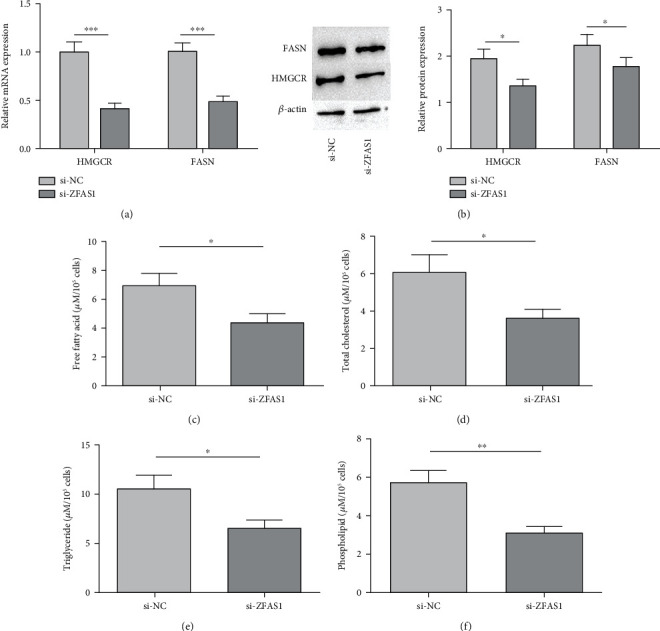
ZFAS1 participates in PC cell lipometabolism. (a) HMGCR mRNA expression; (b) HMGCR protein expression; (c) free fatty acid content in cells; (d) total cholesterol content in cells; (e) triglyceride content in cells; (f) phospholipid content in cells; ^∗^*P* < 0.05;  ^∗∗^*P* < 0.01, and^∗∗∗^*P* < 0.001.

qRT-PCR results identified noticeably decreased mRNA and protein of HMGCR and FASN in si-ZFAS1-transfected BxPC-3 (*P* < 0.05, Figures 3(a) and 3(b)). Then, we tested three typical lipids (CHOLs, TGs, and PLs) in BxPC-3 cells and found remarkably decreased contents of FFAs, TC, TGs, and PLs following ZFAS1 knockout (*P* < 0.05, Figures 3(c)–3(f)).

### 3.4. ZFAS1 Maintains HMGCR mRNA Stabilization by Binding to U2AF2

**Figure 4:**
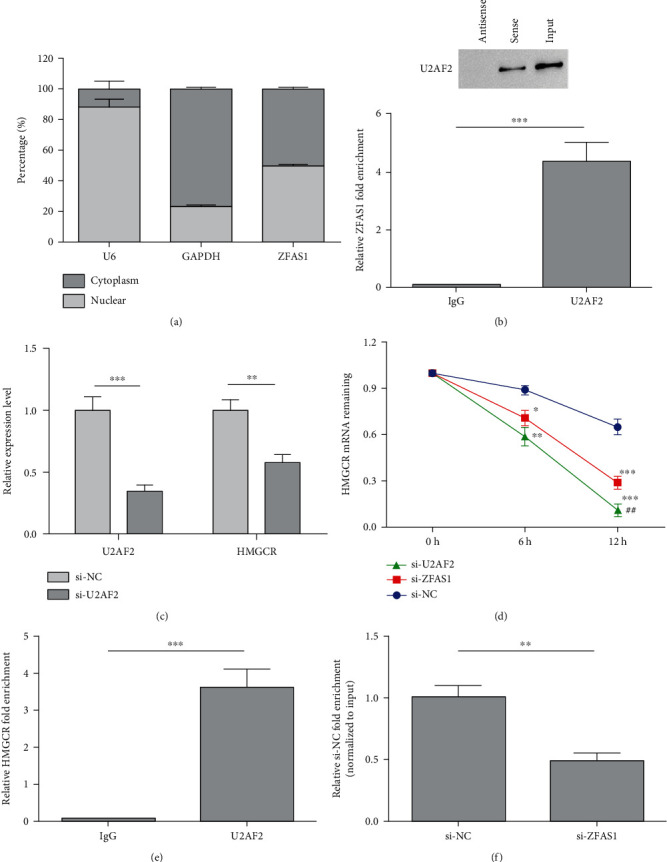
ZFAS1 maintains HMGCR mRNA stabilization by binding to U2AF2. (a) ZFAS1 distribution in BxPC-3 cells; (b) immunoprecipitation protein levels and RIP assay; (c) U2AF2 and HMGCR expression detected by qRT-PCT; (d) HMGCR mRNA stabilization; (e) HMGCR fold enrichment in U2AF2 relative to IgG; (f) qRT-PCR analysis of HMGCR in RIP experience; ^∗^*P* < 0.05;  ^∗∗^*P* < 0.01; ## *P* < 0.01;  ^∗∗∗^*P* < 0.001.

Next, we investigated through which way ZFAS1 modulates HMGCR. ZFAS1 distribution was first determined in BXPC-3 cells and found its abundant expression in the cytoplasm and nucleus (Figure 4(a)). In the cytoplasm, lncRNAs can influence protein activity by interacting with proteins or RNAs. RNA pulldown and RIP results showed that ZFAS1could bind to U2AF2 in BxPC-3 cells (Figure 4(b)). Via qRT-PCR, we found decreased HMGCR in BxPC-3 cells by inhibiting U2AF2 (Figure 4(c)). Then, we treated si-ZFAS1 and si-U2AF2-transfected BxPC-3 cells with ActD for different periods to detect the attenuation of HMGCR mRNA so as to verify that ZFAS1 and U2AF2 can influence HMGCR mRNA stabilization. ZFAS1 and U2AF2 downregulation could lead to a decreased half-life of HMGCR mRNA (Figure 4(d)). Meanwhile, RIP assay results showed that U2AF2 could bind to HMGCR mRNA (Figure 4(e)). Finally, to further confirm whether U2AF2 interacts with ZFAS1 to promote HMGCR mRNA stabilization, we conducted RIP experiments in BxPC-3 cells with either si-ZFAS1 or si-NC transfection. It showed reduced interaction between U2AF2 and HMGCR mRNA by downregulating ZFAS1 in BxPC-3 cells (Figure 4(f)).

### 3.5. Impact of U2AF2 on PC Cell Biological Function

**Figure 5:**
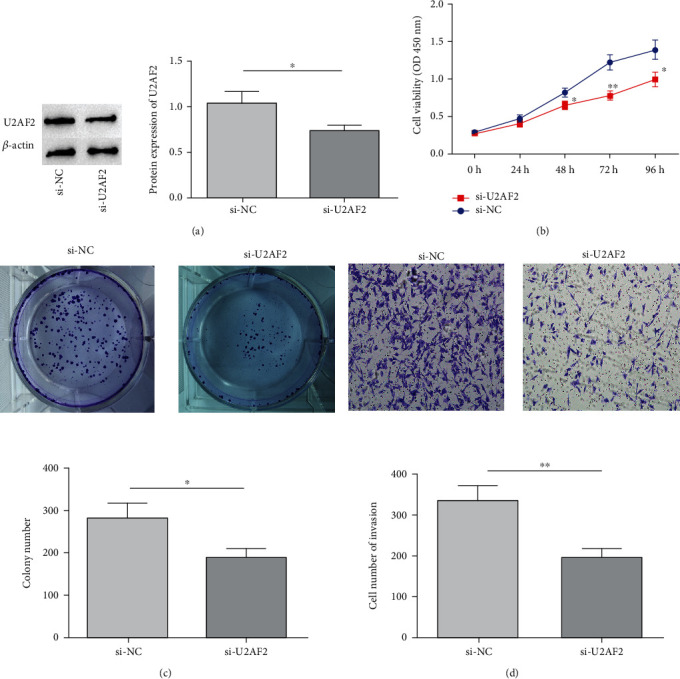
Impact of U2AF2 on biological function of PC cells. (a) U2AF2 expression; (b) cell viability detection; (c) plate clonal cell formation; (d) cell invasiveness; ^∗^*P* < 0.05;  ^∗∗^*P* < 0.01.

The biological function of U2AF2 in BxPC-3 cells was investigated. According to qRT-PCR analysis, U2AF2 decreased noticeably in BxPC-3 cells with si-U2AF2 transfection (*P* < 0.05, Figure 5(a)). Besides, BxPC-3 viability (*P* < 0.05, Figure 5(b)) and clone formation number and invasiveness (*P* < 0.05, Figures 5(c)–5(d)) decreased notably following si-U2AF2 transfection, as indicated by CCK-8 and plate-clone formation results.

### 3.6. Impact of U2AF2 on PC Cell Biological Function

**Figure 6:**
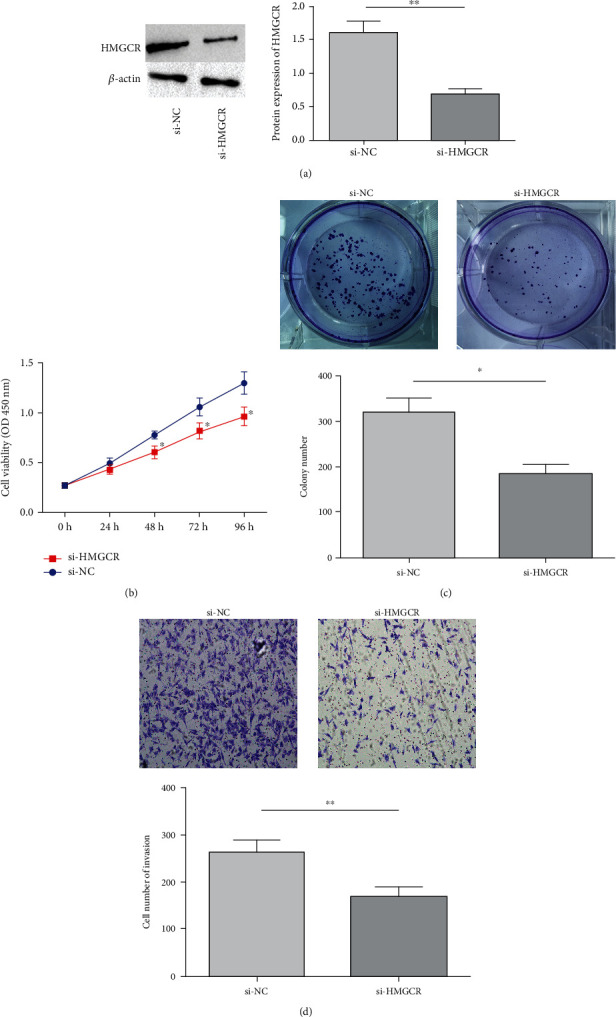
Impact of HMGCR on biological function of PC cells. (a) HMGCR expression; (b) cell viability detection; (c) plate clonal cell formation; (d) cell invasiveness; ^∗^*P* < 0.05;  ^∗∗^*P* < 0.01.

In this part, HMGCR's biological function in BxPC-3 cells was explored. According to qRT-PCR analysis, HMGCR expression decreased noticeably in BxPC-3 cells with si-U2AF2 transfection (*P* < 0.05, Figure 6(a)). HMGCR knockdown also notably reduced BxPC-3 viability and the number of cell clone formation and cell invasiveness (*P* < 0.05, Figures 6(b)–6(d)).

## 4. Discussion

In the tumor microenvironment, tumor cells' nutrition availability constantly changes as the tumor progresses, and cancer cells use lipometabolism to sustain their rapid proliferation, survival, migration, invasiveness, and metastasis [39]. A cancer hallmark has been increasingly identified as a reprogrammed FA metabolism characterized by increased fat production [40]. Enhanced fat production includes de novo FA synthesis [41] and CHOL biosynthesis [42]. Recently, there has been renewed interest in studying lipid reprogramming pathways in tumor cells [43]. But further research is required, given the incomplete understanding of the mechanisms of enhanced FA and CHOL synthesis in tumor cells.

Meanwhile, lncRNAs are critical in cell metabolism by reprogramming tumor cell metabolic pathways [44]. And via integrating vicious transformation and metabolic reprogramming of cells, they regulate a variety of metabolic enzymes [45]. Reportedly, lncRNA HAGLROS regulates lipometabolism reprogramming in intrahepatic cholangiocarcinoma via mTOR axis. ZFAS1 is upregulated in multiple cancers, including gastric [46], cervical [47], and pancreatic carcinomas [48]. This study found upregulated ZFAS1 in PC tissues and cell strains, and ZFAS1 gene knockout statistically suppressed PC cell multiplication and invasiveness *in vitro.*

Similarly, Liu et al. [49] found that lncRNA ZFAS1 promoted pancreatic adenocarcinoma metastasis via sponge aspiration of the miR-3924-mediated RHOA/ROCK2 pathway. The existing studies focus on the involvement of ZFAS1 in the multiplication and metastasis of cancer cells by regulating miRNA-mediated pathways [50, 51], while herein, we reveal a novel role of ZFAS1 and the potential molecule mechanism in PC. We found that ZFAS1 can affect PC lipometabolism, but its underlying mechanism remains to be clarified. Decreased HMGCR expression was observed after knocking out ZFAS1 in PC cells, which made us speculate that ZFAS1 could modulate HMGCR and reduce FFAs, TC, TGs, and PLs in PC cells. HMGCR, the rate-limiting enzyme synthesized by CHOL, is reported to be upregulated in gastric cancer, thus promoting the malignant phenotype of cancer cells [52].

Moreover, HMGCR interferes with cisplatin resistance of ovarian cancer cells, and inhibiting its expression has antimetastasis and antitumor effects [53]. Our study also found that inhibiting HMGCR can inhibit PC cell multiplication and invasiveness. Therefore, we believe that ZFAS1 promotes PC cell growth via upregulating HMGCR to promote CHOL biosynthesis. Subsequently, we found that ZFAS1 does not directly regulate HMGCR expression but through binding to U2AF2 protein through RNA pulldown assay. Further, it was found that downregulating ZFAS1 and U2AF2 expression reduced HMGCR mRNA stabilization in PC cells, and ZFAS1 knockout reduced the interplay of U2AF2 with HMGCR mRNA. Previous studies have shown that lncRNA ZFAS1 further regulates mRNA expression by binding to proteins. lncRNA ZFAS1, for example, promoted colorectal cancer adipogenesis by stabilizing SREBP1 mRNA through binding to polyadenosine binding protein 2 [54]. Similarly, this study found for the first time that lncRNA ZFAS1 enhanced HMGCR mRNA stabilization via binding to U2AF2 to participate in PC lipometabolism, thus regulating the progression of PC. In addition to this, lncRNA SNHG1 and RNA binding protein hnRNPL were found to form a complex and coregulate CDH1 to enhance prostate cancer growth and metastasis [55]. A similar mechanism was presented in our study. In Palangat et al.'s study [56], U2AF, a splicing factor and a heterodimer of U2AF1 and U2AF2, performs the recognition and binding to the 3′ splice site, which is a key initiation step in spliceosome assembly [57]. U2AF1 interacts with its binding partner, U2AF2, to bind to mature RNAs in the cytoplasm and acts as a translational repressor, directly interacting with hundreds of spliced and polyadenylated mRNAs in the cytoplasm.

## 5. Conclusion

To summarize, we discovered abnormally high lncRNA ZFAS1 expression in PC, and ZFAS1 increased PC cell multiplication and invasiveness by regulating lipometabolism. Its main mechanism of action is that ZFAS1 binds to U2AF2 and promotes its interaction with HMGCR mRNA to reprogram lipometabolism, thus promoting CHOL synthesis and ultimately promoting PC cell growth. However, this study also has some shortcomings. For example, the effect of ZFAS1 on PC through this pathway has not been verified in vivo and its mechanism in vivo is still unknown. Therefore, we will conduct further in vivo validation of its mechanism of action in subsequent studies. We collectively believe that ZFAS1 is a promising diagnostic marker for PC, and its mechanism of affecting lipometabolism reprogramming can provide a new direction and target for PC treatment.

## Figures and Tables

**Table 1:** Primer sequences utilized in this study.

Genes	Primer sequence
ZFAS1	Forward (F): 5′-GCTATTGTCCTGCCCGTTAG-3′
	Reverse (R): 5′-TCGTCAGGAGATCGAAGGTT-3′
U2AF2	F: 5′-ATGACCCCTGACGGTCTGG-3′
	R: 5′-GAGCGGAACTCCAAAAAGGC-3′
HMGCR	F: 5′-TTCTTGCCAACTACTTCGTGTT-3′
	R: 5′-GCTGCCAAATTGGACGACC-3′
FASN	F: 5′- CCGAGACACTCGTGGGCTA-3′
	R: 5′-CTTCAGCAGGACATTGATGCC-3′
GAPDH	F: 5′-AGCCACATCGCTCAGACAC-3′
	R: 5′-GCCCAATACGACCAAATCC-3′
*β*-Actin	F: 5′-GTCATTCCAAATATGAGATGCGT-3′
	R: 5′-GCATTACATAATTTACACGAAAGCA-3′

## Data Availability

The labeled dataset used to support the findings of this study is available from the corresponding author upon request.

## Abbreviations

PC: Pancreatic carcinoma
IncRNAs: Long-chain noncoding RNAs
TCGA: The Cancer Genome Atlas
GEPIA: Gene Expression Profiling Interactive Analysis
CAS: Chinese Academy of Sciences
HPNE: Human pancreatic ductal epithelial cells
CHOL: Cholesterol
DMEM: Dulbecco's modified Eagle medium
PCR: Polymerase chain reaction
SDS-PAGE: Sodium Dodecyl Sulphate-Polyacrylamide Gel Electrophoresis
RIPA buffer: Radioimmunoprecipitation assay buffer
CV: Crystal violet
PBS: Phosphate-buffered saline
Tc: Total cholesterol
TGs: Triglycerides
PLs: Phospholipids
FA: Fatty acid
FFA: Free fatty acid
ActD: Actinomycin D
PAAD: Pancreatic adenocarcinoma database
GTEx: The Genotype-Tissue Expression.

## References

[1] Sung H., Ferlay J., Siegel R. L. (2021). Global cancer statistics 2020: Globocan estimates of incidence and mortality worldwide for 36 cancers in 185 countries. *CA: a Cancer Journal for Clinicians*.

[2] Deplanque G., Demartines N. (2017). Pancreatic cancer: are more chemotherapy and surgery needed?. *The Lancet*.

[3] Park W., Chawla A., O’Reilly E. M. (2021). Pancreatic Cancer. *JAMA*.

[4] Saad K. (2014). Childhood epilepsy: an update on diagnosis and management. *American Journal of Neuroscience*.

[5] Kindler H. L. (2018). A glimmer of hope for pancreatic cancer. *New England Journal of Medicine*.

[6] Maan M., Peters J. M., Dutta M., Patterson A. D. (2018). Lipid metabolism and lipophagy in cancer. *Biochemical and Biophysical Research Communications*.

[7] Li H., Feng Z., He M.-L. (2020). Lipid metabolism alteration contributes to and maintains the properties of cancer stem cells. *Theranostics*.

[8] Pompeia C., Lopes L., Miyasaka C., Procopio J., Sannomiya P., Curi R. (2000). Effect of fatty acids on leukocyte function. *Brazilian Journal of Medical and Biological Research*.

[9] Jarc E., Petan T. (2019). lipid droplets and the management of cellular stress. *The Yale Journal of Biology and Medicine*.

[10] Bian X., Liu R., Meng Y., Xing D., Xu D., Lu Z. (2021). Lipid metabolism and cancer. *Journal of Experimental Medicine*.

[11] Broadfield L. A., Pane A. A., Talebi A., Swinnen J. V., Fendt S.-M. (2021). Lipid metabolism in cancer: new perspectives and emerging mechanisms. *Developmental Cell*.

[12] Clerc P., Bensaadi N., Pradel P., Estival A., Clemente F., Vaysse N. (1991). Lipid-dependent proliferation of pancreatic cancer cell lines. *Cancer Research*.

[13] Chen M., Zhang J., Sampieri K. (2018). An aberrant srebp-dependent lipogenic program promotes metastatic prostate cancer. *Nature Genetics*.

[14] Fhu C. W., Ali A. (2020). Fatty acid synthase: an emerging target in cancer. *Molecules*.

[15] Swierczynski J., Hebanowska A., Sledzinski T. (2014). Role of abnormal lipid metabolism in development, progression, diagnosis and therapy of pancreatic cancer. *World Journal of Gastroenterology: WJG*.

[16] Bhan A., Soleimani M., Mandal S. S. (2017). Long noncoding rna and cancer: a new paradigm. *Cancer Research*.

[17] Ghafouri-Fard S., Fathi M., Zhai T., Taheri M., Dong P. (2021). Lncrnas: novel biomarkers for pancreatic cancer. *Biomolecules*.

[18] Zhou C., Yi C., Yi Y. (2020). Lncrna pvt1 promotes gemcitabine resistance of pancreatic cancer via activating wnt/*β*-catenin and autophagy pathway through modulating the mir-619-5p/pygo2 and mir-619-5p/atg14 axes. *Molecular Cancer*.

[19] He J., Li F., Zhou Y. (2020). LncRNA xloc_006390 promotes pancreatic carcinogenesis and glutamate metabolism by stabilizing c-myc. *Cancer Letters*.

[20] Chen L.-L. (2016). Linking long noncoding rna localization and function. *Trends in Biochemical Sciences*.

[21] Heindel J. J., Blumberg B., Cave M. (2017). Metabolism disrupting chemicals and metabolic disorders. *Reproductive Toxicology*.

[22] Yi S.-Y., Hao Y.-B., Nan K.-J., Fan T.-L. (2013). Cancer stem cells niche: a target for novel cancer therapeutics. *Cancer Treatment Reviews*.

[23] Nilsson A., Nielsen J. (2017). Genome scale metabolic modeling of cancer. *Metabolic Engineering*.

[24] Lu W., Cao F., Wang S., Sheng X., Ma J. (2019). Lncrnas: the regulator of glucose and lipid metabolism in tumor cells. *Frontiers in Oncology*.

[25] Shang C., Wang W., Liao Y. (2018). Lnmicc promotes nodal metastasis of cervical cancer by reprogramming fatty acid metabolism. *Cancer Research*.

[26] Askarian-Amiri M. E., Crawford J., French J. D. (2011). Snord-host RNAZfas1is a regulator of mammary development and a potential marker for breast cancer. *RNA*.

[27] Ye D., Jian W., Feng J., Liao X. (2018). Role of long noncoding rna zfas1 in proliferation, apoptosis and migration of chondrocytes in osteoarthritis. *Biomedicine & Pharmacotherapy*.

[28] Hu F., Shao L., Zhang J., Zhang H., Wen A., Zhang P. (2020). Knockdown of zfas1 inhibits hippocampal neurons apoptosis and autophagy by activating the pi3k/akt pathway via up-regulating mir-421 in epilepsy. *Neurochemical Research*.

[29] Tang X., Yin R., Shi H. (2020). Lncrna zfas1 confers inflammatory responses and reduces cholesterol efflux in atherosclerosis through regulating mir-654-3p-adam10/rab22a axis. *International Journal of Cardiology*.

[30] Ma K., Zhang L. (2021). Overview: lipid metabolism in the tumor microenvironment. *Lipid Metabolism in Tumor Immunity*.

[31] Will C. (2006). Spliceosome structure and function. *The RNA world*.

[32] Wahl M. C., Will C. L., Lührmann R. (2009). The spliceosome: design principles of a dynamic rnp machine. *Cell*.

[33] Jurica M. S., Moore M. J. (2003). Pre-mrna splicing: awash in a sea of proteins. *Molecular Cell*.

[34] Zhang J., Manley J. L. (2013). Misregulation of pre-mrna alternative splicing in cancer. *Cancer Discovery*.

[35] Stamm S. (2002). Signals and their transduction pathways regulating alternative splicing: a new dimension of the human genome. *Human Molecular Genetics*.

[36] Chen L., Weinmeister R., Kralovicova J. (2017). Stoichiometries of u2af35, u2af65 and u2 snrnp reveal new early spliceosome assembly pathways. *Nucleic Acids Research*.

[37] Shirai C. L., Ley J. N., White B. S. (2015). Mutant u2af1 expression alters hematopoiesis and pre-mrna splicing in vivo. *Cancer Cell*.

[38] Yoshida K., Sanada M., Shiraishi Y. (2011). Frequent pathway mutations of splicing machinery in myelodysplasia. *Nature*.

[39] Zhao S., Cheng L., Shi Y., Li J., Yun Q., Yang H. (2021). Mief2 reprograms lipid metabolism to drive progression of ovarian cancer through ros/akt/mtor signaling pathway. *Cell Death & Disease*.

[40] Menendez J. A., Lupu R. (2017). Fatty acid synthase (fasn) as a therapeutic target in breast cancer. *Expert Opinion on Therapeutic Targets*.

[41] Kuzu O. F., Noory M. A., Robertson G. P. (2016). The role of cholesterol in cancer. *Cancer Research*.

[42] Cheng C., Geng F., Cheng X., Guo D. (2018). Lipid metabolism reprogramming and its potential targets in cancer. *Cancer Communications*.

[43] Tan Y. T., Lin J. F., Li T., Li J. J., Xu R. H., Ju H. Q. (2021). Lncrna-mediated posttranslational modifications and reprogramming of energy metabolism in cancer. *Cancer Communications*.

[44] Xu Y., Qiu M., Shen M. (2021). The emerging regulatory roles of long non-coding rnas implicated in cancer metabolism. *Molecular Therapy*.

[45] Ma J., Feng J., Zhou X. (2020). Long non-coding RNA HAGLROS regulates lipid metabolism reprogramming in intrahepatic cholangiocarcinoma via the mTOR signaling pathway. *Experimental and Molecular Pathology*.

[46] Xu W., He L., Li Y., Tan Y., Zhang F., Xu H. (2018). Silencing of lncrna zfas1 inhibits malignancies by blocking wnt/*β*-catenin signaling in gastric cancer cells. *Bioscience, Biotechnology, and Biochemistry*.

[47] Feng L.-L., Shen F.-R., Zhou J.-H., Chen Y.-G. (2019). Expression of the lncrna zfas1 in cervical cancer and its correlation with prognosis and chemosensitivity. *Gene*.

[48] Rao M., Xu S., Zhang Y., Liu Y., Luan W., Zhou J. (2021). Long non-coding RNA ZFAS1 promotes pancreatic cancer proliferation and metastasis by sponging miR-497-5p to regulate HMGA2 expression. *Cell Death & Disease*.

[49] Liu J., Zhu Y., Ge C. (2020). Lncrna zfas1 promotes pancreatic adenocarcinoma metastasis via the rhoa/rock2 pathway by sponging mir-3924. *Cancer Cell International*.

[50] Su Y., Hou W., Zhang C. (2022). Long non-coding rna zfas1 regulates cell proliferation and invasion in cervical cancer via the mir-190a-3p/klf6 axis. *Bioengineered*.

[51] Zhang F., Li Y., Xu W., He L., Tan Y., Xu H. (2019). Long non-coding rna zfas1 regulates the malignant progression of gastric cancer via the microrna-200b-3p/wnt1 axis. *Bioscience, Biotechnology, and Biochemistry*.

[52] Chushi L., Wei W., Kangkang X., Yongzeng F., Ning X., Xiaolei C. (2016). Hmgcr is up-regulated in gastric cancer and promotes the growth and migration of the cancer cells. *Gene*.

[53] Zhao J., Zhang X., Gao T. (2020). Sik2 enhances synthesis of fatty acid and cholesterol in ovarian cancer cells and tumor growth through pi3k/akt signaling pathway. *Cell Death & Disease*.

[54] Wang H., Chen Y., Liu Y. (2022). The lncrna zfas1 regulates lipogenesis in colorectal cancer by binding polyadenylate-binding protein 2 to stabilize srebp1 mrna. *Molecular Therapy-Nucleic Acids*.

[55] Tan X., Chen W.-B., Lv D.-J. (2021). LncRNA SNHG1 and RNA binding protein hnRNPL form a complex and coregulate CDH1 to boost the growth and metastasis of prostate cancer. *Cell Death & Disease*.

[56] Palangat M., Anastasakis D. G., Fei D. L. (2019). The splicing factor u2af1 contributes to cancer progression through a noncanonical role in translation regulation. *Genes & Development*.

[57] Merendino L., Guth S., Bilbao D., Martínez C., Valcárcel J. (1999). Inhibition of _msl-2_ splicing by Sex-lethal reveals interaction between U2AF^35^ and the 3 ′ splice site AG. *Nature*.

